# Supplementary material for: TDP-43 loss induces extensive cryptic polyadenylation in ALS/FTD
Source: bioRxiv. 2024 Jan 23:2024.01.22.576625. Preprint. [Version 1] doi: 10.1101/2024.01.22.576625 (PMC10836071; doi:10.1101/2024.01.22.576625)
Supplement: Supplement 3 [file NIHPP2024.01.22.576625v1-supplement-3.pdf]

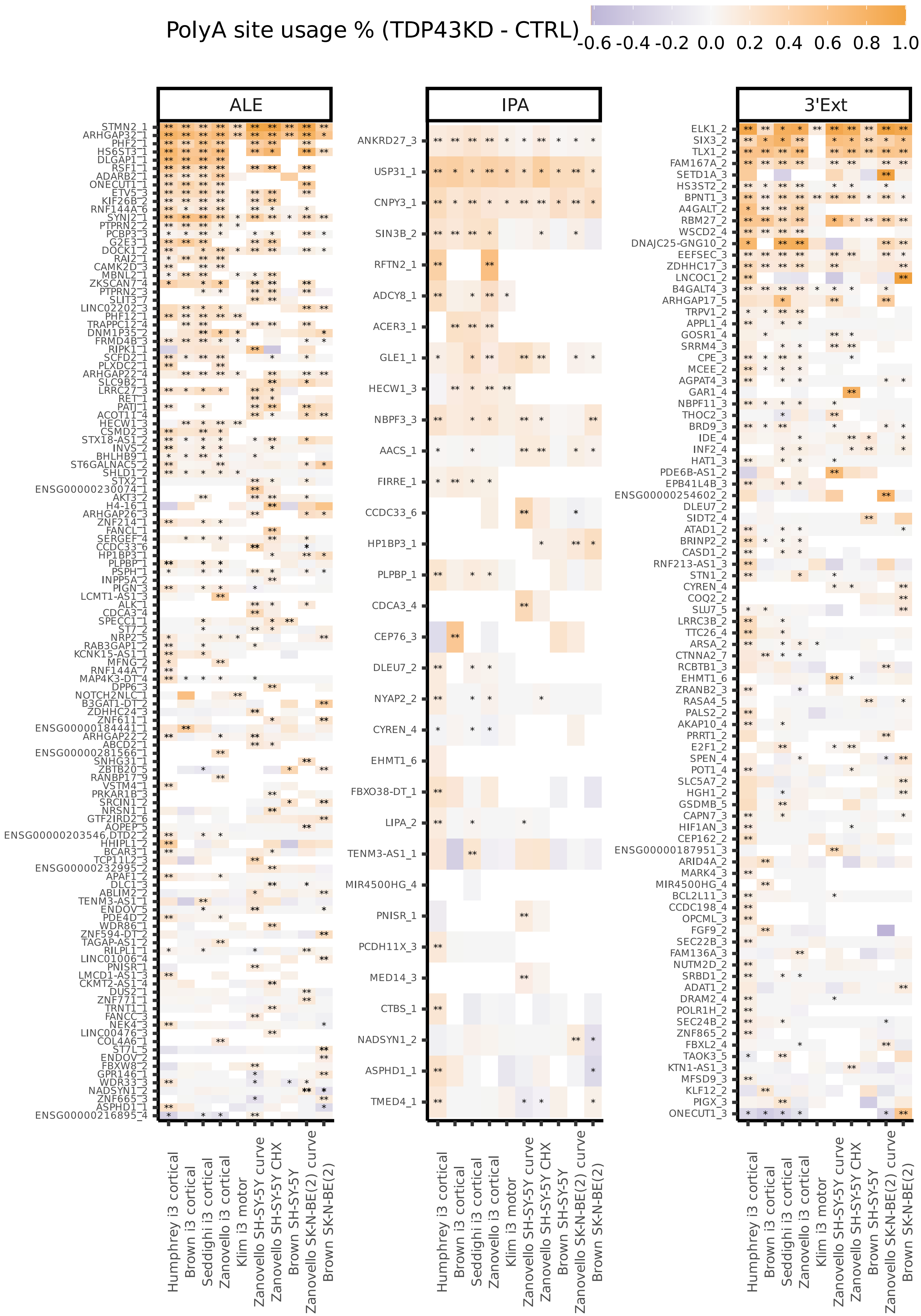

PolyA site usage % (TDP43KD - CTRL)
-0.6 -0.4 -0.2 0.0 0.2 0.4 0.6 0.8 1.0
ALE
IPA
3'Ext
Humphrey i3 cortical
Brown i3 cortical
Seddighi i3 cortical
Zanovello i3 cortical
Klim i3 motor
Zanovello SH-SY-5Y curve
Zanovello SH-SY-5Y CHX
Brown SH-SY-5Y
Zanovello SK-N-BE(2) curve
Brown SK-N-BE(2)

bioRxiv preprint doi: https://doi.org/10.1101/2024.01.22.576625; this version posted January 23, 2024. The copyright holder for this preprint (which was not certified by peer review) is the author/funder, who has granted bioRxiv a license to display the preprint in perpetuity. It is made available under aCC-BY 4.0 International license.

Supplementary Figure 1 - Consistency of response to TDP-43 depletion across compendium of in-vitro datasets

Differential usage of cryptic APA events across the compendium of in-vitro datasets. Cells are coloured in accordance to magnitude and direction of change in usage, where positive values (orange) indicate increased usage in TDP-43 knockdown ('TDP43KD') samples. Blank cells indicate the event was not expressed at sufficient levels to be assessed for differential usage. Rows are sorted in decreasing order of the sum of $-\log_{10}$ transformed p-values weighted by the change in usage between TDP-43 knockdown and control samples (TDP43KD - CTRL) in each dataset. A single asterisk indicates that the isoform was considered significantly regulated in a dataset (Benjamini-Hochberg adjusted p-value < 0.05), and two asterisks indicate the isoform is considered cryptic in a given dataset.

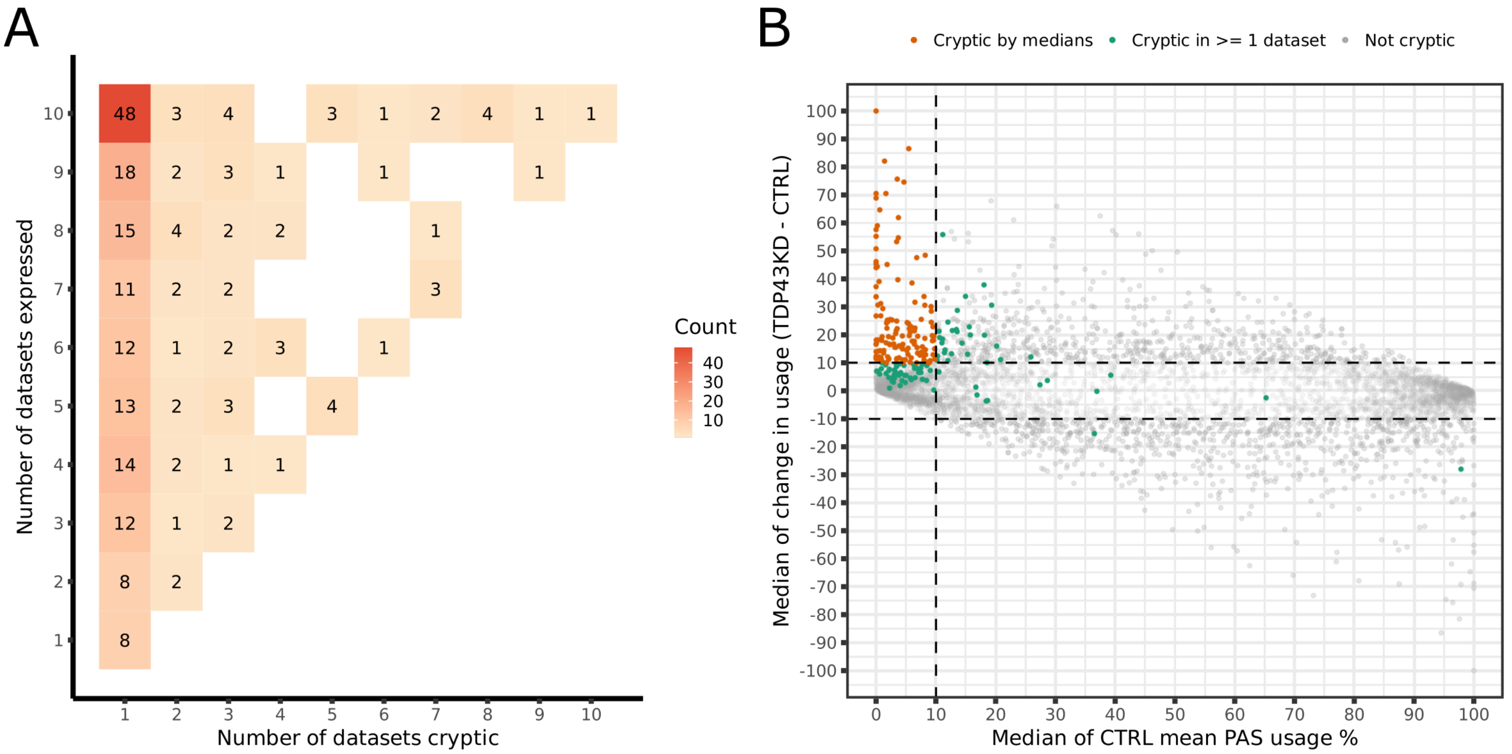

Supplementary Figure 2

bioRxiv preprint doi: https://doi.org/10.1101/2024.01.22.576625; this version posted January 23, 2024. The copyright holder for this preprint (which was not certified by peer review) is the author/funder, who has granted bioRxiv a license to display the preprint in perpetuity. It is made available under aCC-BY 4.0 International license.

Supplementary Figure 2 - Consistency of cryptic status across compendium of *in vitro* datasets

A) Relationship between the number of datasets in which APAs are called cryptic and their detection. Count labels indicate the number of unique cryptic APAs that fall into the given bin. Events are considered expressed if they pass minimum expression criteria to be evaluated for differential isoform usage (Methods).

B) Last exons responsive to TDP-43 depletion. All points represent a last exon passing a Benjamini-Hochberg adjusted p-value < 0.05 threshold in at least one dataset. Where a last exon passes the threshold in multiple datasets, the median values across datasets are calculated to represent the basal usage and change in usage upon TDP-43 depletion. Points that pass cryptic expression criteria in at least one dataset but pass (orange) or fail (green) the criteria when calculating the median change in usage and expression in control (CTRL) cells across datasets with an Benjamini-Hochberg adjusted p-value < 0.05 are highlighted.

A

AA-containing motifs	GUGUGA, AAUGAA, GAAUGA, UGAAUG, AUGAAU, GUGAAU, GAAUGU, UUGAAU
YA-containing motifs	AUGUGU, GUAUGU, GUGUAU, UGUGUA, UGUAUG, UGCAUG
YG-containing motifs	UGUGUG, GUGUGU, UGUGCG, UGCGUG, CGUGUG, GUGUGC
Combined motifs	All of above

B

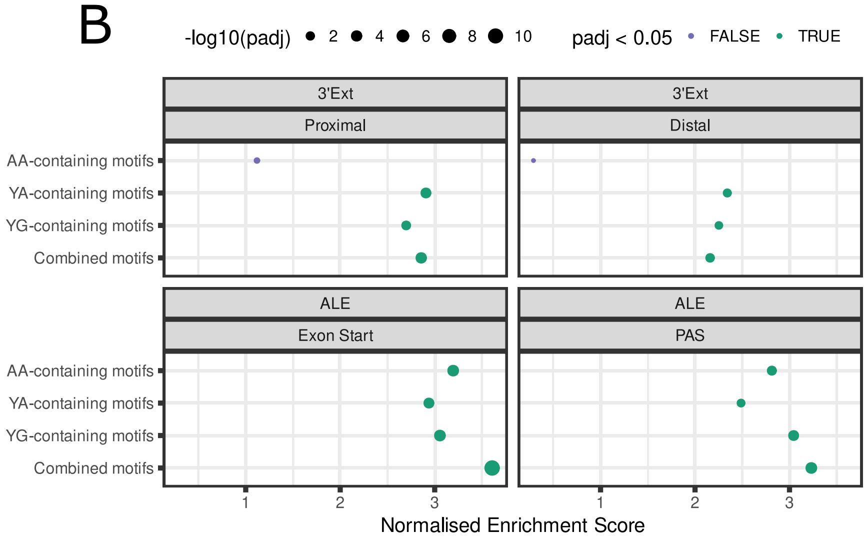

Supplementary Figure 3

bioRxiv preprint doi: https://doi.org/10.1101/2024.01.22.576625; this version posted January 23, 2024. The copyright holder for this preprint (which was not certified by peer review) is the author/funder, who has granted bioRxiv a license to display the preprint in perpetuity. It is made available under aCC-BY 4.0 International license.

Supplementary Figure 3 - Enrichment of previously defined TDP-43 binding hexamers at cryptic APA boundaries

A) Table listing previously defined TDP-43 hexamer groups[6]. 'Y' codes for a pyrimidine nucleotide.

B) Gene set enrichment analysis (GSEA) of enriched TDP-43 binding 6mers on de-novo enriched 6-mers around cryptic landmarks. The panels and labels correspond to regions evaluated for iCLIP binding as in Fig. 1D . The area of the points is proportional to the $-\log_{10}$ transformed adjusted p-value (adjusted with respect to all region types and motif groups), and the colour denotes whether the Benjamini-Hochberg adjusted p-value passes (green) or fails (purple) a significance threshold of < 0.05.

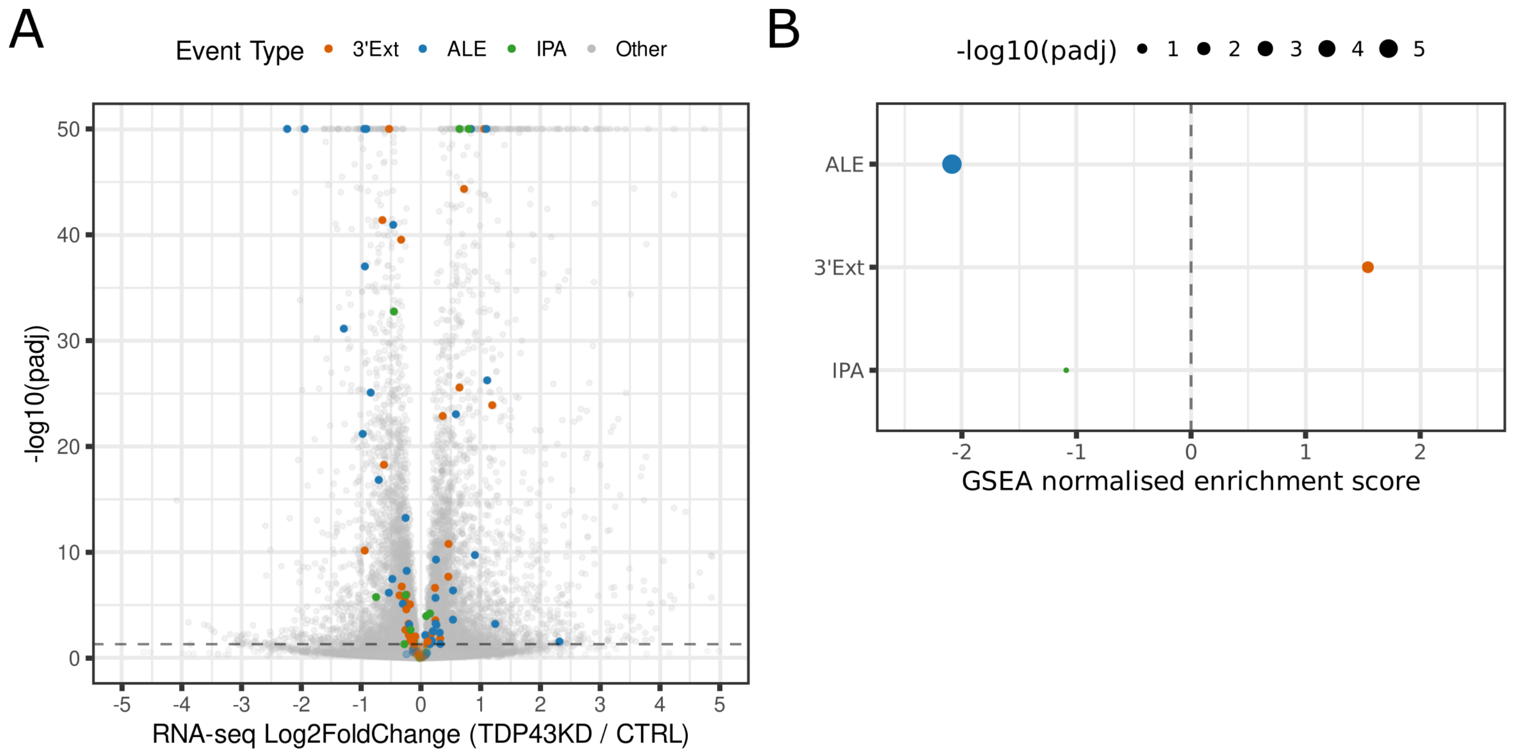

Supplementary Figure 4

bioRxiv preprint doi: https://doi.org/10.1101/2024.01.22.576625; this version posted January 23, 2024. The copyright holder for this preprint (which was not certified by peer review) is the author/funder, who has granted bioRxiv a license to display the preprint in perpetuity. It is made available under aCC-BY 4.0 International license.

Supplementary Figure 4 - Analysis of cryptic APA categories in Ribo-seq data

A) Volcano plot of differential expression analysis of RNA-seq data between TDP-43 knockdown (TDP43KD) and control (CTRL) i3Neurons. Cryptic APA genes with significant differential expression (Benjamini-Hochberg adjusted p-value < 0.05) are highlighted in orange (3'Ext), blue (ALE) or green (IPA). Genes with a -$\log_{10}$ transformed Benjamini-Hochberg adjusted p-value greater than 50 are collapsed to 50 for visualisation purposes.

B) Gene Set Enrichment Analysis (GSEA) of cryptic APA categories in i3Neuron Ribo-seq differential expression fold change ranks. The area of the points is proportional to the -$\log_{10}$ transformed Benjamini-Hochberg adjusted p-value. Points are coloured according to their APA category as in A).
